# Effects of Acute Laboratory Stress on Executive Functions

**DOI:** 10.3389/fpsyg.2016.00461

**Published:** 2016-03-31

**Authors:** Katrin Starcke, Carina Wiesen, Patrick Trotzke, Matthias Brand

**Affiliations:** ^1^Department of General Psychology: Cognition, University of Duisburg-EssenDuisburg, Germany; ^2^Erwin L. Hahn Institute for Magnetic Resonance ImagingEssen, Germany

**Keywords:** stress, executive functions, attention and inhibition, task management, planning, monitoring, coding

## Abstract

Recent research indicates that stress can affect executive functioning. However, previous results are mixed with respect to the direction and size of effects, especially when considering different subcomponents of executive functions. The current study systematically investigates the effects of stress on the five components of executive functions proposed by Smith and Jonides (1999): attention and inhibition; task management; planning; monitoring; and coding. Healthy participants (*N* = 40) were either exposed to the computerized version of the Paced Auditory Serial Addition Test as a stressor (*N* = 20), or to a rest condition (*N* = 20). Stress reactions were assessed with heart rate and subjective measures. After the experimental manipulation, all participants performed tasks that measure the different executive functions. The manipulation check indicates that stress induction was successful (i.e., the stress group showed a higher heart rate and higher subjective responses than the control group). The main results demonstrate that stressed participants show a poorer performance compared with unstressed participants in all executive subcomponents, with the exception of monitoring. Effect sizes for the tasks that reveal differences between stressed and unstressed participants are high. We conclude that the laboratory stressor used here overall reduced executive functioning.

## Introduction

Executive functions are cognitive control mechanisms that allow goal-oriented, flexible, and effective acting and thinking (Baddeley and Hitch, 1974; Lezak, 1995), which are essential for our everyday functioning. However, so far there is no consensus on the sub-functions, which should be subsumed under the term executive functions (Eslinger et al., 1996; Stuss and Alexander, 2000). There is a debate whether one central factor underlies all executive functions (de Frias et al., 2006) or whether separate functions are modular in nature (Godefroy et al., 1999). Miyake et al. (2000) examined the three executive functions shifting, updating, and inhibition. They concluded that these functions are distinguishable, but also correlated (see also Miyake and Friedman, 2012). Furthermore, they acknowledge the existence of further executive functions that can be regarded as subcomponents of the three functions studied (e.g., monitoring) or that contain the interplay of all three functions (e.g., planning). According to the definition by Smith and Jonides (1999), executive functions include attention and inhibition, task management, planning, monitoring, and coding. Attention and inhibition cover the direction of attention toward relevant information, whereas irrelevant information is ignored. Furthermore, actions that are dominant, but irrelevant to the current situation are inhibited. Task management includes that tasks and processes are sorted and that one can switch between them. Planning allows reaching a certain goal by dividing tasks into subtasks. Monitoring covers the controlling and updating of information with the help of working memory contents. Coding means that information is transferred to the working memory for time and place of appearance. The current empirical study addresses these five subcomponents of executive functions according to Smith and Jonides (1999). Each component is assessed by one neuropsychological test. On a neural level, the prefrontal cortex, particularly the dorsolateral section, is supposed to be a key brain region that is involved in these functions (Goldman-Rakic, 1996; Smith and Jonides, 1999). However, other brain regions such as the thalamus and the basal ganglia are also involved in executive functioning (Jurado and Rosselli, 2007).

A factor that can affect executive functioning is stress. Stress occurs when a demand exceeds the regulatory capacity of an organism (Dickerson and Kemeny, 2004; Koolhaas et al., 2011). Stress elicits psychological, physiological, and behavioral reactions, but individuals react differently toward stressors (Kudielka et al., 2009). On a neural level, stress affects functioning of prefrontal cortex regions that have a high density of stress hormone receptors (Arnsten, 2009). During early stress responses, a salience network is triggered that provides resources for immediately recognizing and reacting toward threats. At the same time, the executive control network that enables the usage of higher order cognitive processes is decreased (Hermans et al., 2014). This executive control network is important for long-term survival. It has been suggested that executive functions are the first cognitive functions that suffer when people are stressed (Diamond, 2013).

Recent research examined the effects of acute laboratory stress on subsequent executive performance in humans. There is increasing evidence that stress can decrease attention and inhibition (Scholz et al., 2009; Henderson et al., 2012; Sänger et al., 2014) although effects are not found in all studies (Starcke et al., 2008). Studies that assessed the other four components of executive functions under acute laboratory stress are scarce so far and provided mixed results. Task management operationalized with dual-task paradigms were found to be improved under stress (Beste et al., 2013) while other studies did not find any enhancing or deteriorating effects of stress (Pabst et al., 2013; Gathmann et al., 2014). Task management operationalized with a switching task was found to be impaired under stress (Plessow et al., 2012a). Most of the studies cited assessed one or two components of executive functioning, only. The aim of the current study is to investigate the effect of acute stress compared to rest condition on all five components of executive functions that were proposed by Smith and Jonides (1999) systematically. We hypothesize that stress decreases executive functioning in accordance with Hermans et al. (2014). For exploratory reasons, we also calculate the interaction of age and stress on executive performance. Old age shows adverse effects on executive functioning (review in Harada et al., 2013) and a recent study demonstrated that stress exposure and executive functioning were negatively related in older adults (Roiland et al., 2015).

## Materials and Methods

### Participants

Forty healthy participants (20 females) were included in the analysis. Exclusion criteria were chronic or acute diseases, psychological problems and color blindness. Participants’ age ranged from 20 to 67 years (mean = 44.05, *SD* = 17.31). Half of the participants were young adults (19–35 years old) and the other half were older adults (56–67 years old) Most of them (*N* = 35) had an education level comparable to a high school degree. They were assigned randomly to the stress or the control condition. The stress group and the control group did not differ concerning gender distribution (10 males and 10 females in each group), age (*t* = 0.22, *df* = 38, *p* = 0.83) and level of education (*U* = 196.5, *p* = 0.92). Results demonstrate successful randomization. The study was approved by the local ethics committee and all participants provided written informed consent and were not paid for their participation.

### Methods

#### Stress Induction and Control Condition

To induce stress in the stress group, the computerized version of the Paced Auditory Serial Addition Test (PASAT-C; Lejuez et al., 2003) was used. In this task, participants have to add numbers that are serially presented on a screen as fast as possible. If the answer is wrong or given too late an aversive sound occurs. There are three levels with increasing difficulty (shorter inter-stimulus interval) and increasing level-length (from 3–10 min). However, the third level can be quit by the participants. The PASAT-C or modified versions reliably elicit autonomic arousal and cortisol stress responses were also observed (Lejuez et al., 2003; Mathias et al., 2004; Reinhardt et al., 2012). Participants of the control condition were asked to relax instead of performing the PASAT-C for approximately the same duration as the PASAT-C.

#### Stress Measurement

The state version of the State Trait Anxiety Inventory (STAI; Spielberger, 1972) was used to measure anxiety before and after the stress induction and after the tests of executive functions were completed (points of measurement 1, 2, and 3). The questionnaire consists of 20 items that measure current anxiety on a four point Likert scale from 1 (not at all) to 4 (very much). Item scores are summed up (after inverting items that are pooled differently) and can thus range from 20 to 80.

Heart rate was measured in beats per minute during a baseline period, during stress induction/control condition, and during the executive tasks (also named points of measurement 1, 2, and 3, although points represent segments in which the heart rate was averaged). The Polar RS800CX system (Polar Electro, Kempele, Finnland) was used to acquire heart rate. The system includes a sensor with embedded electrodes in a belt (worn across the chest and placed above the xiphoid process), which detects cardiac electrical impulses. The sensor transmitted the detection of these impulses to the receiver and the interbeat interval was converted to the heart rate in beats per minute. Previous studies demonstrated that the acquisition of the heart rate with Polar systems and a sampling rate of 5 s (i.e., the interbeat interval is recorded at this epoch) is valid and comparable to R-wave peaks measures of electrocardiograms (Goodie et al., 2000). Nevertheless, we choose a much more precise sampling rate of 2 s in order to maximize accuracy. At the beginning of each segment (baseline period, stress induction/control condition, executive tasks) a marker was set to the data and the mean hart rate was analyzed for each segment with the Polar Trainer 5 software. Heart rate increases during stress were calculated by using delta scores (mean heart rate during the PASAT-C minus mean heart rate at baseline).

#### Tasks Measuring Executive Functions

##### Attention and inhibition

The Color-Word-Interference-Test after Stroop (CWIT; Bäumler, 1985) was used to assess attention and inhibition. The task consists of three parts that have to be performed as fast as possible. In the first part, participants have to read aloud a list of color words (red, yellow, green, and blue). In the second part, they have to name the color of printed rectangles (also red, yellow, green, and blue). The third part is the interference part. Participants have to name the colors of colored printed words. However, each color word is printed in a color that is not consistent with the word. For example, the word “blue” is printed in red and participants have to ignore the word blue and only have to name the color red. Thus, participants have to pay attention to the color and inhibit the urge to read the word.

##### Task management

To assess task management, the Trail Making Test (TMT; Reitan, 1958) was used. The test consists of two parts, A and B, that both have to be performed as fast as possible. In part A, circles with numbers from 1 to 25 are spread over a sheet of paper. They have to be linked with a pen in ascending order (1, 2, 3, and so on). In part B, circles with numbers from 1 to 12 and letters of the alphabet from A to K are spread over a sheet of paper. They have to be linked with a pen in ascending and alternating order (1, A, 2, B, 3, C and so on). Thus, in part B of the test the participants have to manage the switching between the alphabet and the numbers.

##### Planning

To assess planning, the Tower of Hanoi (ToH; Simon, 1975) was used in the computerized five disk version. In this task, participants face a model with three pegs. On one of these pegs, there are five disks sorted from largest to smallest (the largest on the ground and the smallest on top). Participants are told to move these five disks to another peg according to certain rules. They can only move one disk at a time and only smaller disks can be placed on larger disks. This has to be done as fast as possible. Time until task completion and the number of moves are analyzed. Thus, participants have to plan their moves according to sub-goals.

##### Monitoring

The Balance Switch Task (BST; Schiebener et al., 2014) was used to measure monitoring abilities. The task consists of two sets (A and B). In each of the sets participants have two tasks. In set A, numbers from 01 to 99 are presented. Task 1 is to indicate whether the number is odd or even and task 2 is to indicate whether the number is below or above 50. In set B, geometrical figures are presented. Task 1 is to indicate whether the diagonal hedging of the figure is directed to the upper left or the upper right corner and task 2 is to indicate whether the figure is presented horizontally or vertically. Thus, overall, participants have four tasks and they can switch between them voluntary. They are instructed to work on each task as equally often as possible, classify the stimuli as correctly as possible, and to work as fast as possible. However, switching between the sets results in a loss of time and participants are unaware of the complete task duration. The BST is performed twice each in a block of 4 min. The main outcome measure is the deviation from balance (not performing the tasks equally). Thus, participants have to monitor which task to perform how long.

##### Coding

The Digit Substitution Symbol Test (DSST; Wechsler, 1981) was used for the assessment of Coding. Participants are presented a list of digit-symbol pairs and a list of 67 digits alone. They should write the corresponding symbol below the digit. They should work as fast as possible to complete as many symbols as possible within the prescribed time limit of 90 s. Thus, participants have to code which digit represents which symbol.

#### Procedure

After participants signed written informed consent, heart rate measurement started. During the baseline period, participants filled out questionnaires concerning sociodemography and exclusion criteria. This lasted about 5 min. Then the first STAI was filled out and the experimental manipulation (PASAT-C or rest condition, ∼20 min) started. The second STAI was filled out and then the BST (∼20 min) was performed. After the BST, the TMT, the CWIT, the ToH, and the DSST (each lasting a few minutes) were performed in randomized order. Finally, the third STAI was filled out and participants were fully debriefed and thanked for participation.

#### Statistical Analysis

Data were analyzed with SPSS version 22 (IBM, Armonk, NY, USA). Heart rate was analyzed with a repeated measurement ANOVA with point of measurement as within subject factor and group as between subject factor. The same was done for the STAI. Partial eta squared ($\eta_{p}^{2}$) was used as a measure of effect size. Furthermore, heart rate and STAI values of the single points of measurement were compared between groups with *t*-tests for independent samples. Between-group differences in age were also analyzed with *t*-tests. Between-group differences of educational level were analyzed with the Mann–Whitney *U* test. All measures of executive functions were analyzed with *t*-tests for independent samples. Cohens *d* was used as a measure of effect size. Bonferroni correction (for 16 comparisons) was applied for the tests of executive functions. Pearson correlations were performed for the stress condition between the stress responses and executive performance. We used moderated regression analyses to test the interaction between stress induction and age on executive performance. Stress induction was the nominal predictor (yes or no), age was the continuous moderator, and the respective executive measure was the dependent variable.

## Results

### Level of Stress

Prior to stress exposure, the stressed participants did not show higher heart rate (*M* = 78.15, *SD* = 6.33) compared with the control participants (*M* = 78.20, *SD* = 7.13). During stress exposure, the stressed participants showed higher heart rate (*M* = 97.30, *SD* = 7.35) than the control participants (*M* = 78.35, *SD* = 5.39) and differences were significant (*t* = 9.30, *df* = 38, *p* < 0.001). During the executive tasks, there was a marginal significant higher heart rate in the stress group (*M* = 80.85, *SD* = 5.83) than in the control group (*M* = 77.70, *SD* = 4.12) during the BST (*t* = 1.97, *df* = 38, *p* = 0.056), while this difference was no longer observed during the other executive tasks.

On a subjective level, participants of the stress group did not report higher anxiety (*M* = 48.55, *SD* = 3.12) compared to unstressed participants (*M* = 47.50, *SD* = 2.50) prior to stress exposure. After stress exposure, participants of the stress group reported higher anxiety levels (*M* = 49.00, *SD* = 3.23) than the control participants (*M* = 46.35, *SD* = 2.35) and differences were significant (*t* = 2.97, *df* = 38, *p* = 0.005). After performing the executive tasks, participants of the stress group (*M* = 46.45, *SD* = 3.09) still reported higher anxiety than the control participants (*M* = 44.45, *SD* = 3.36) and differences were marginally significant (*t* = 1.96, *df* = 38, *p* = 0.057).

The 2 (group) × 3 (points of measurement) repeated measures ANOVAs indicate significant effects for heart rate and partially for subjective anxiety. Results of the ANOVAs are shown in **Table 1**.

**Table 1:** Indicators of stress.

Stress indicator	*F*	*df*	*MSE*	*P*	$\eta_{p}^{2}$
Heart rate: Time	105.69	2, 76	13.38	<0.001	0.74
Heart rate: Group	15.38	1, 38	85.53	<0.001	0.29
Heart rate: Time × Group	85.40	2, 76	13.38	<0.001	0.69
STAI: Time	10.35	2, 76	7.54	<0.001	0.21
STAI: Group	9.58	1, 38	11.30	<0.005	0.20
STAI: Time × Group	0.86	2, 76	7.54	0.43	0.02


*MSE, mean standard error; STAI, state trait anxiety inventory.*

### Executive Performance

Results indicate that stressed participants performed worse compared with unstressed participants in nearly all executive domains that were assessed. Results are presented separately for the speed component (time in seconds or number of correct answers within a time limit) and the accuracy component (number of mistakes, number of steps needed, or balance score). In the CWIT, the TMT and the ToH the stressed participants needed more time for task completion than the unstressed participants. In the DSST, stressed participants made fewer correct substitutions within the time limit than unstressed participants. However, this finding does not remain significant after Bonferroni correction. Nevertheless, the difference between stressed and unstressed participants had a large effect size. In the CWIT, the TMT and the DSST the stressed participants made more mistakes than the unstressed participants. In the ToH, stressed participants needed more moves than the unstressed participants. Differences do not remain significant after Bonferroni correction in the ToH and the DSST. Nevertheless, the difference between stressed and unstressed participants had large effect sizes. The only task in which stressed and unstressed participants’ performance did not differ at all is the BST. Thus, monitoring is the only component in which stressed participants did not perform worse than unstressed participants, whereas attention and inhibition, task management, planning, and coding were deteriorated under stress. Results are summarized in **Tables 2** and **3**.

**Table 2:** Results of the executive tasks (speed).

Test	SG *M* (*SD*)	CG *M* (*SD*)	*t*	*df*	*P*	*d*
CWIT reading	35.11 (3.02)	29.11 (2.41)	6.93	38	<0.001	2.20
CWIT naming	46.69 (2.54)	40.14 (2.34)	8.48	38	<0.001	2.68
CWIT interference	76.69 (4.06)	69.55 (2.75)	6.51	38	<0.001	2.06
TMT-A	32.69 (2.29)	25.54 (2.30)	9.86	38	<0.001	3.12
TMT-B	70.12 (4.19)	50.52 (3.82)	15.46	38	<0.001	4.89
ToH	190.91 (13.28)	136.89 (15.33)	11.91	38	<0.001	3.77
DSST correct	59.75 (2.12)	61.35 (1.46)	–2.78	38	<0.01	0.88


*SG, stress group; CG, control group; CWIT, Color Word Interference Test; TMT, Trail Making Test; ToH, Tower of Hanoi; DSST, Digit Symbol Substitution Test. All results represent time in seconds except results of the DSST. For the DSST, the correct answers within the time limit are presented.*

**Table 3:** Results of the executive tasks (accuracy and balance).

Test	SG *M* (*SD*)	CG *M* (*SD*)	*t*	*df*	*P*	*d*
CWIT reading	1.65 (0.81)	0.40 (0.60)	5.54	38	<0.001	1.75
CWIT naming	22.75 (3.39)	5.65 (3.27)	16.26	38	<0.001	5.14
CWIT interference	33.45 (3.58)	17.40 (1.76)	18.01	27.69	<0.001	5.70
TMT-A	2.55 (1.00)	0.35 (0.59)	8.49	30.73	<0.001	2.68
TMT-B	3.45 (0.94)	0.70 (0.80)	9.93	38	<0.001	3.14
ToH moves	48.75 (7.07)	41.35 (7.88)	3.13	38	<0.005	0.99
DSST	7.25 (2.12)	5.65 (1.46)	2.78	38	<0.01	0.88
BST block 1	0.47 (0.26)	0.41 (0.08)	0.85	22.61	0.41	0.31
BST block 2	0.39 (0.24)	0.41 (0.12)	–0.24	28.10	0.81	0.10


*SG, stress group; CG, control group; CWIT, Color Word Interference Test; TMT, Trail Making Test; ToH, Tower of Hanoi; DSST, Digit Symbol Substitution Test; BST, Balance Switch Task. Results of the CWIT, TMT, and DSST represent the number of mistakes. Results of the ToH represent the number of moves. For the BST, the deviation from balance is presented.*

### Check for Potential Outliers

**FIGURE 1:**
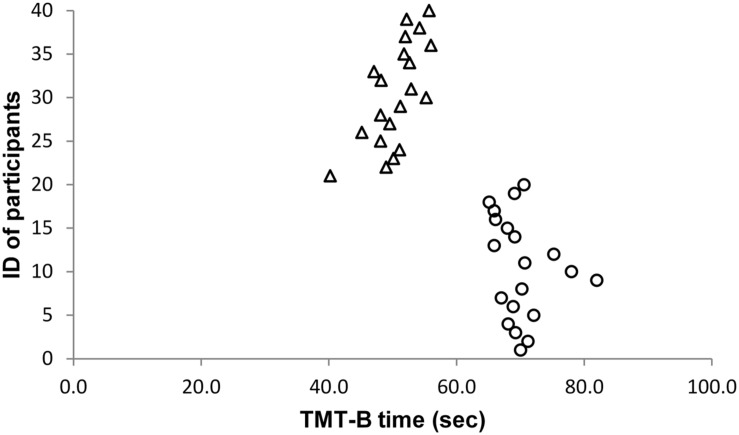
**Trail Making Test (TMT)**.

**FIGURE 2:**
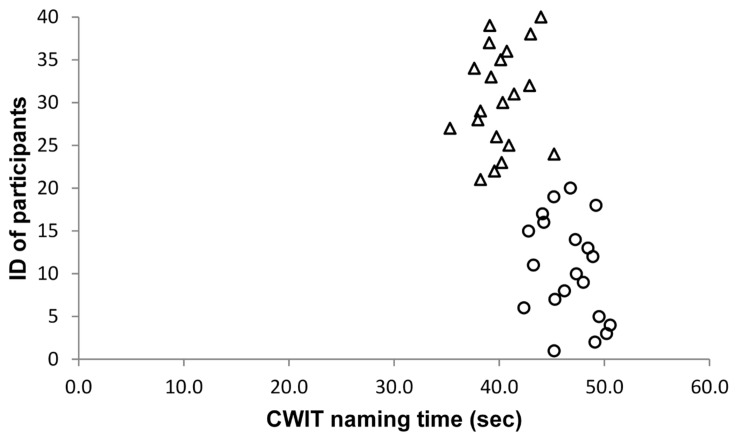
**Color Word Interference Test (CWIT)**.

**FIGURE 3:**
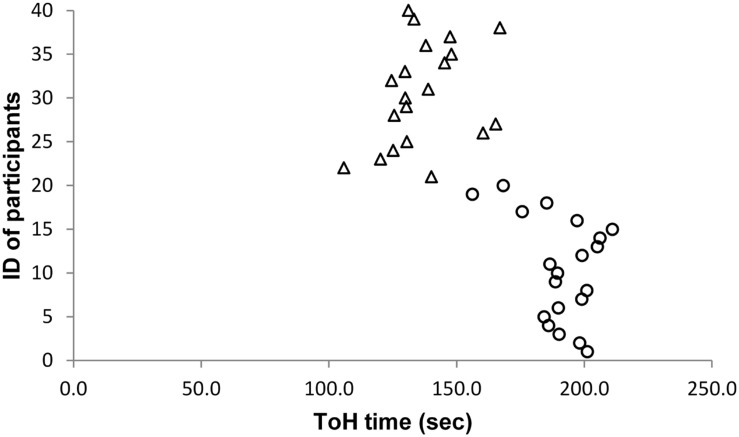
**Tower of Hanoi (ToH)**.

**FIGURE 4:**
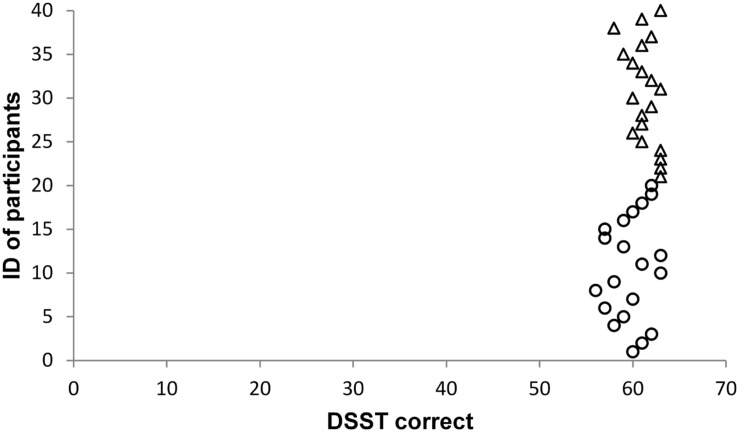
**Digit Symbol Substitution Test (DSST)**.

**FIGURE 5:**
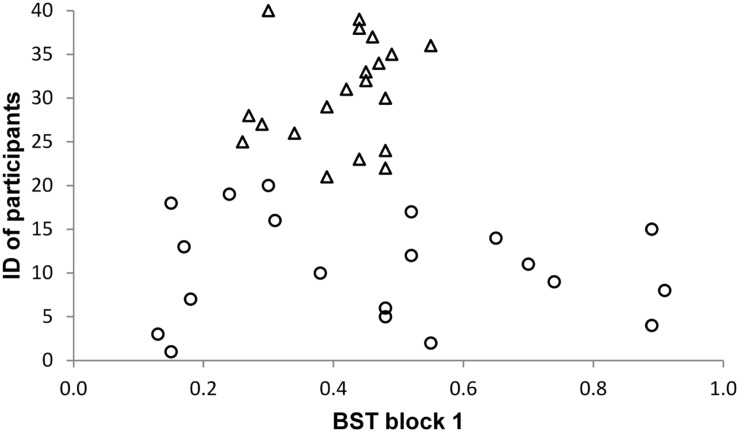
**Balance Switch Task (BST)**.

As the effect sizes of significant results are very large, we carefully inspected whether there might be outliers in our sample. However, we did not find any abnormalities in our data. **Figures 1–5** show the participants’ results for one dependent variable of each executive task. Participants with the IDs 1 to 20 are in the stress group (represented by circles), participants with the IDs 21 to 40 are in the control group (represented by triangles). Results of the TMT-B indicate that all participants of the control group were faster compared with participants of the stress group.

### Relationship between Stress Responses and Executive Functioning

In the stress group, we analyzed the relationship between the heart rate increase (heart rate during the PASAT-C minus heart rate at baseline) and the performance in the executive tasks. However, none of the executive task performances were related to the heart rate increase (all *p*s > 0.05). The same was done for the subjective stress reactions (STAI after the PASAT-C minus STAI at baseline). However, no significant results were observed either (all *p*s > 0.05).

### Moderating Effects of Age

Moderated regressions have been performed to test a potential interacting effect of stress and age on executive functions with group (stress versus control) as predictor and age as moderator. However, no clear pattern could be observed. Interactions between stress and age were only found with the TMT-B as dependent variable (changes in *R*^2^ = 0.035, *F*(1,36) = 12.61, *p* = 0.001) and with the second block of the BST (changes in *R*^2^ = 0.29, *F*(1,36) = 16.22, *p* < 0.001). In the TMT-B, the older participants performed better than the younger participants within the stress group, while in the control group the younger participants performed better than the older participants. In the second block of the BST, older participants in the stress group performed poorer than younger participants in the stress group, but in the control group, older participants performed better than younger participants.

## Discussion

Results support the hypothesis that stress can impair executive functioning. All subcomponents were performed significantly poorer in the stress group than in the control group, with monitoring being the only exception. Results were similar for the speed component and the accuracy component. As a necessary precondition for interpreting the results, the induction of stress was successful as indicated by heart rate and partially by subjective anxiety responses. No consistent moderating effects of age were observed.

Results are in line with the assumption that the executive control network is decreased under stress (Hermans et al., 2014). As an underlying mechanism, impaired prefrontal cortex functioning due to the release of stress hormones is suggested. The prefrontal cortex (Goldman-Rakic, 1996; Smith and Jonides, 1999) and particularly the dorsolateral prefrontal cortex (Nee et al., 2013) is known as a key region for executive functioning. It has numerous receptors to which stress hormones can bind and stress leads to alterations in neural activations (Dedovic et al., 2009; Pruessner et al., 2010). Animal studies suggest that stress-induced elevations of stress hormones impair neurons’ capacity to maintain persistent spiking activity (Devilbiss et al., 2012). Some recent human behavioral studies also suggest that stress impairs executive functioning (Scholz et al., 2009; Henderson et al., 2012; Plessow et al., 2012a; Sänger et al., 2014) although contradicting results were also reported (e.g., Beste et al., 2013). Studies that assessed executive functions after stress induction and neural reactions during the performance of executive tasks (others than working memory tasks) in humans are, to the best of our knowledge, still missing.

In the current study, all executive functions but monitoring were performed worse in the stress group than in the control group. Effect sizes are very large and we therefore carefully inspected if there were outliers in our data, but we did not find any. There is a notable dissociation between monitoring and the other four components of executive functions. Reasons for this dissociation have to be elucidated. Recent studies suggest a shift from serial to parallel information processing after the induction of stress (Plessow et al., 2012b; Pabst et al., 2013; Gathmann et al., 2014) and this may allow parallel goal monitoring. Parallel goal monitoring might prevent from deteriorating stress effects on monitoring assessed with the BST. In the BST, participants have to keep in mind parallel tasks and this ability might be unimpaired by stress because information is processed in a parallel manner anyway.

Furthermore, the BST itself might induce some level of moderate stress. This has been reported by some participants, but the physiological data do not confirm that. Nevertheless, a moderate level of subjective stress during the BST even in the control group might have interfered with group differences in the stress versus the control group. Another reason for different results for monitoring and the other executive tasks might be that the timing between stress induction and assessment of executive components differed from one another. The BST was always performed immediately after stressor cessation, because the task took relatively long (20 min) compared to the other tasks and a complete randomization of the tasks would have resulted in an inconsistent position of all four other tasks. The four other tasks were performed after the BST in randomized order. Immediately after the cessation of stress, a marginally increased heart rate could be observed. Thus, during the BST, a stress response of the sympathetic system can be assumed. During the other executive tasks, no elevated heart rate in the stress group could be observed, indicating that sympathetic system stress responses returned to baseline during the other executive tasks. This is astonishing because the BST is unimpaired by stress induction in spite of a sympathetic system stress response, while the other tasks are impaired although no sympathetic system stress response could be observed during these tasks. However, the stressor as well as the BST lasted approximately 20 min meaning that all executive tasks were performed within a time period in which substantial cortisol stress responses could be assumed. Cortisol secretion has some latency, but lasts a long period after cessation of stress (Dickerson and Kemeny, 2004). In a modified PASAT-C this temporal pattern of cortisol secretion was also observed (Reinhardt et al., 2012). That means we can assume substantial cortisol stress responses during all executive tasks. Nevertheless, time related dynamics of stress responses that might affect executive tasks cannot be ruled out here.

Therefore, not counterbalancing completely all executive tasks can be viewed as a limitation of the current study. A further limitation is that cortisol was not directly measured. Particularly cortisol stress responses appear to be related to cognitive performance, such as working memory (Schoofs et al., 2008) or decision making (Leder et al., 2013). As cortisol was not assessed in the current study, such relationships could not be investigated here. In addition, cortisol stress responses are influenced by many factors such as smoking status, recent meal time, recent exercise, recent intake of medication, hormonal contraceptives and menstrual cycle in females (review in Kudielka et al., 2009). It is therefore not possible to conclude that stress induced cortisol secretion caused the decline in executive performance in the stressed participants. A relationship between the individual heart rate increase of stressed participants and executive tasks could not be observed, so a potential link between biological stress responses and reduced executive performance cannot be established with the current data. The questionnaire that was administered to assess subjective stress does not directly measure stress, but anxiety (Ekkekakis, 2013) and should be replaced by questionnaires that are more sensitive to stress induced changes in future studies. A main methodological shortcoming of the current study is that the control group only relaxed while the stress group was exposed to a cognitive stressor. Although this procedure is not completely uncommon in stress research (e.g., Starcke et al., 2008), it might have been better to administer a standardized cognitive task in the control group which does not elicit stress. As the stress group was exposed to a cognitive challenge and potentially experienced anger and frustration, other mechanisms than stress might have been influenced upcoming executive performance, for example cognitive depletion (Baumeister et al., 1998; Schmeichel et al., 2003).

In future studies, a standardized control protocol should be applied to the participants of the control group. In addition, the five components of executive functions could be assessed at baseline, and a second time after stress induction. Such a repeated-measures design would help to minimize potential confounding variables. Furthermore, cortisol responses should be assessed in order to establish a direct relationship between executive performance and individual stress responses. A recent study indicates that good executive functions prevent from very high stress reactions (Hendrawan et al., 2012). Thus, in larger studies one could investigate the reciprocal relationship between stress reactions and executive functioning together with potential moderating variables of stress reactions and task performance.

## Author Contributions

KS designed the study and wrote the manuscript; CW designed the study, collected the data, and analyzed the data; PT supervised the heart rate data collection and analysis and revised the manuscript; MB designed the study and revised the manuscript.

## Conflict of Interest Statement

The authors declare that the research was conducted in the absence of any commercial or financial relationships that could be construed as a potential conflict of interest.
